# Effects of Nitrogen Deficiency on the Metabolism of Organic Acids and Amino Acids in *Oryza sativa*

**DOI:** 10.3390/plants11192576

**Published:** 2022-09-29

**Authors:** Ling-Hua Chen, Zu-Xin Cheng, Ming Xu, Zhi-Jian Yang, Lin-Tong Yang

**Affiliations:** 1 College of Jinshan, Fujian Agriculture and Forestry University, Fuzhou 350002, China; 2 Fujian Engineering Technology Research Center of Breeding and Utilization for Special Crops, College of Agriculture, Fujian Agriculture and Forestry University, Fuzhou 350002, China; 3 Key Laboratory of Crop Biotechnology, College of Agriculture, Fujian Agriculture and Forestry University, Fuzhou 350002, China; 4 College of Resources and Environment, Fujian Agriculture and Forestry University, Fuzhou 350002, China

**Keywords:** rice, nitrogen deficiency, organic acid, nitrogen metabolism, growth

## Abstract

Organic acids metabolism and nitrogen (N) metabolism in rice seedlings and the relationship between them are not fully understood. In this study, rice (*Oryza sativa* L. ssp. Indica) variety “Huanghuazhan” was used as the experimental material, and three N levels (5 mM, 1 mM, and 0 mM NH_4_NO_3_) were set by the hydroponic method for different levels of N treatment. Our results showed that the increased content of malate in rice leaves caused by reducing N level was related to the increased synthesis of malate (the activity of leaf PEPC increased)and the decreased degradation of malate (the activity of leaf NADP-ME decreased), while the increased contents of citrate and isocitrate in rice leaves caused by reducing N level might not be caused by the increased biosynthesis, but due to the decrease in degradation of citrate and isocitrate (the activities of leaf CS, ACO, and NADP-IDH decreased). The increased content of malate in rice roots caused by reducing N level might be related to the increased biosynthesis and the decreased degradation of root malate (the activities of root NAD-MDH and PEPC increased, while the activity of NADP-ME decreased). Compared to the control (5 mM NH_4_NO_3_), the increased content of citrate in rice roots caused by reducing N level might be related to the increased biosynthesis rather than the decreased degradation of citrate, due to the higher activities of CS and ACO in rice roots under 0 mM N and 1mM N treatment when compared to that of the control ones. At the same time, the increased content of isocitrate in roots was related to the increased isomerization of isocitrate (the activity of root ACO increased) and the decreased degradation of isocitrate (the activity of root NADP-IDH decreased). With the reducing N level, the activities of N metabolism-related enzymes, such as nitrate reductase (NR), glutamine synthetase (GS), and glutamate synthase (GOGAT), decreased in rice leaves and roots, resulting in the decreased contents of total free amino acids (TFAAs) and soluble proteins in rice seedlings, and finally led to the growth inhibition. Our results showed that the dynamics of organic acids metabolism caused by reducing N level were different in rice leaves and roots. In conclusion, there was a close correlation between organic acids metabolism and N metabolism in rice leaves and roots under N-limited conditions; furthermore, such a correlation was more obvious in rice leaves than that of roots.

## 1. Introduction

Nitrogen (N) is a macro-nutrient for plant growth and development. It is an important component of functional molecules, such as nucleic acid, amino acid, protein, chlorophyll, and some plant hormones. It is one of the main factors limiting plant growth and crop yield [1]. N is the most abundant nutrient element in plants, accounting for about 2–4% of the dry weight of plants [2]. Although some organic forms of N, such as urea, amino acids, and amides, can also be absorbed by plants, ammonium and nitrate are the two main forms of N obtained by plant roots from soil solutions. These N forms are scarce in natural soil. When the total N absorbed by plant roots from the rhizosphere is less than the N required for plant growth, N deficiency will occur. In the absence of artificial N fertilizer input, N deficiency is one of the important factors restricting the crop yield and quality of agricultural products. Organic acids are the intermediate products of major carbon metabolism in plant cells and participate in various biochemical pathways, such as glycolysis, the tricarboxylic acid (TCA) cycle, photorespiration, the glyoxylic acid cycle, or the photosynthetic C4 cycle [3]. The intermediate products of organic acid metabolism, such as oxaloacetic acid and 2-ketoglutarate, provide the carbon skeletons for transamination and amino acid synthesis [4,5,6]. It is important to study the relationship between N level and organic acid metabolism and N metabolism for understanding plant N metabolism and guiding the application of N fertilization.

Application of N fertilizer to the field can regulate the flowering period of rice and increase the number of panicles, the number of grains per panicle, and the rice yield [7], while N deficiency can lead to the degradation of photosynthetic pigments in crop leaves, the yellowing of leaves, the reduction in carbon dioxide assimilation rate, and the inhibitionof the growth and yield of crops. The phenomenon that N deficiency reduces the photosynthetic rate and affects plant growth has been reported in many crops, including tea, citrus, corn, cucumber, sunflower, tobacco, rice, and barley [2,8,9,10,11].

The carbon (C) metabolism and N assimilation are interrelated. N assimilation requires energy and a carbon skeleton, which is provided by carbohydrate metabolism. Nitrate is an important source of inorganic N in plants. Changes in exogenous nitrate concentration could affect the rapid change in plant metabolism. For example, it could induce the synthesis of nitrate-assimilating enzymes and accelerate glycolysis and the TCA cycle to convert starch into organic acids to assimilate ammonium [12]. Excess nitrate may be accumulated into vacuoles, but excessive ammonium absorbed by crop roots and ammonium produced by nitrate through the action of nitrate reductase (NR) and nitrite reductase (NiR) have toxic effects on plants. Ammonium assimilation requires an adequate carbon skeleton and promotes carbon flow into the TCA cycle [13]. Inorganic N is assimilated and incorporated into glutamine, glutamate, asparagine, and aspartic acid, which are important N carriers in plants. These primary amino acids are the main compounds in the total free amino acid pool of many plants and can be used to synthesize other amides, such as urea, other amino acids, and amines [12,13]. In this process, the glutamine synthetase/glutamate synthase (GS/GOGAT) cycle is the main pathway of nitrogen assimilation in plants. GS catalyzes the reaction of NH_4_^+^ with glutamate to produce glutamine. GOGAT subsequently catalyzes the transfer of amide groups from glutamine to 2-ketoglutarate (2-OG), generating two glutamate molecules [14]. Among them, 2-ketoglutarate, the main carbon receptor in the GS/GOGAT pathway, is synthesized through the combined action of phosphoenolpyruvate (PEP) carboxylase (PEPC, EC 4.1.1.31), pyruvate kinase (PK), pyruvate dehydrogenase (PDH), aconitase (ACO, EC 4.2.1.3), and NADP-isocitrate dehydrogenase (NADP-IDH, EC1.1.1.42) [15].

The organic-acid-metabolism-related enzymes PEPC, NAD-malate dehydrogenase (NAD-MDH, EC 1.1.1.37), NADP-malic enzyme (NADP-ME, EC 1.1.1.40), citrate synthase (CS, EC 4.1.3.7), ACO, and NADP-IDH are considered to play an important role in balancing C and N metabolism [16,17]. PEPC catalyzes irreversible β-carboxylation of PEP in the presence of HCO_3_^−^ and Mg_2_^+^ to yield oxaloacetate, which is used to synthesize malate by NAD-MDH [18,19]. The degradation of malate is mediated by cytosolic NADP-ME in plant cells [20,21]. In the cytoplasm and mitochondrion, PEP is converted to acetyl-CoA by the sequential actions of PK and PDH. Aceytl-CoA and oxaloacetate are then combined by CS to generate citrate in the mitochondrion [22]. The citrate is reversibly isomerized to isocitrate via *cis*-aconitate by ACO [23] Isocitrate is then catalyzed to 2OG by NADP-IDH [24]. It was found on the model plant *Arabidopsis thaliana* that the interaction of C-N metabolism could significantly affect the expression levels of more than 300 genes (*p* < 0.05). Among them, the expression levels of PEPC, NAD-MDH, NADP-ME, and NADP-IDH were found to be altered by N deficiency [5]. Studies have shown that plant physiological metabolism, energy, and protein synthesis are found to be significantly affected by the interaction between C/N signals [25,26]. Specific metabolites of C/N metabolism, such as glucose, sucrose, or nitrate, serve as signals to regulate genes encoding enzymes involved in many important biological processes, including photosynthesis, carbon metabolism, nitrogen metabolism, and metabolite allocation [5]. In the study of N-deficiency transcriptome in rice, Shao et al. reported that N deficiency treatment upregulated the expression level of *PEPC* in rice leaves and increased the content of malic acid in leaves [2]. Similarly, it was also found that N-deficiency treatment increased the contents of glycolic acid and succinic acid in tea leaves and citric acid, malic acid, isocitric acid, succinic acid, and fumaric acid in tea roots [9]. 

Rice is an important grain crop in the world. Nitrogen fertilizer plays an important role in its growth and yield [27]. Meanwhile, increasing the soil nitrogen supply can significantly improve the soil carbon pool [28]. Previous studies have shown that under N deficiency, the activities of enzymes related to N metabolism (such as GS, GOGAT, and glutamate dehydrogenase (GDH)) were affected to varying degrees in rice [14]. Therefore, in order to further understand the mode of C and N metabolism in rice under N reduction conditions, this study carried out research on organic acid metabolism and N metabolism of rice leaves and roots under low-N and N-deficiency treatments. The results of this research can be used for rice N management, high yield, and high-quality cultivation and sustainable development of farmland.

## 2. Results

### 2.1. Effects of Different Nitrogen Treatments on Rice Growth and Nitrogen Content in Leaves and Roots

**Figure 1:**
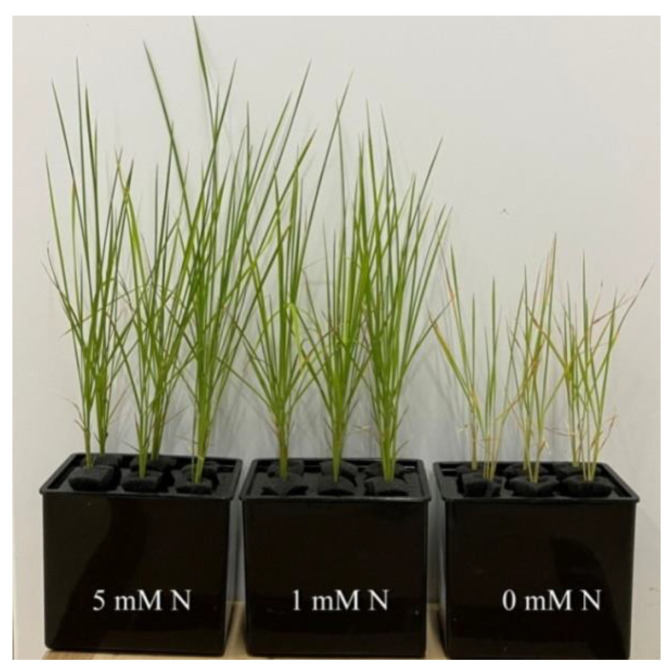
Phenotypeof *O. sativa* seedlings under different N levels. Rice seedlings were transplanted to the Hogland nutrient solution containing 5 mM (control), 1 mM (low N), and 0 mM NH_4_NO_3_ (N deficiency). Seedlings were kept under a 14 h light/10 h dark regime with white photo-illumination of 150 μmol m^−2^ s^−1^ as well as a relative humidity of 68% in a light incubator (28 °C). Fifteen days after transplanting, leaf and root samples were collected.

**Figure 2:**
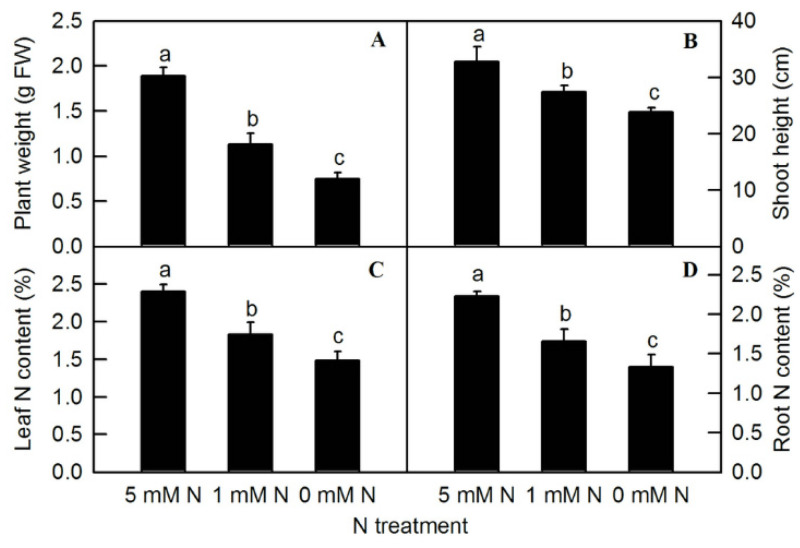
Effects of N deficiency on plant weight (**A**), shoot height (**B**), leaf N content (**C**), and root N content (**D**) in *O. sativa*. Bars represent means ± SD (*n* = 4). Difference among the treatments was analyzed by Duncan’s multiple range test. Different letters above the bar indicate a significant difference at *p* < 0.05.

Different nitrogen levels significantly affected the growth of rice seedlings (Figure 1). Compared to the control (5 mM NH_4_NO_3_), under the treatment of 0 mM NH_4_NO_3_, rice leaves turned yellow and plant growth was inhibited. After the treatment of different N levels, the fresh weight of the plants was measured. Compared to the control, both low-N (1 mM NH_4_NO_3_) and N-deficiency (0 mM NH_4_NO_3_) treatments significantly reduced the fresh weight of rice seedlings (Figure 2A). The shoot height of rice seedlings was measured. Compared to the control, N-deficiency and low-N treatment significantly reduced the shoot height of rice seedlings (Figure 2B). After oven-drying, N content was measured. The result showed that the leaf N and root N of rice seedlings were significantly reduced by either low N (1 mMNH_4_NO_3_) or N deficiency (0 mM NH_4_NO_3_) (Figure 2C,D).

### 2.2. Effects of Different N Treatments on the Contents of Organic Acids, TFAAs, and Soluble Protein in Rice Leaves and Roots

**Figure 3:**
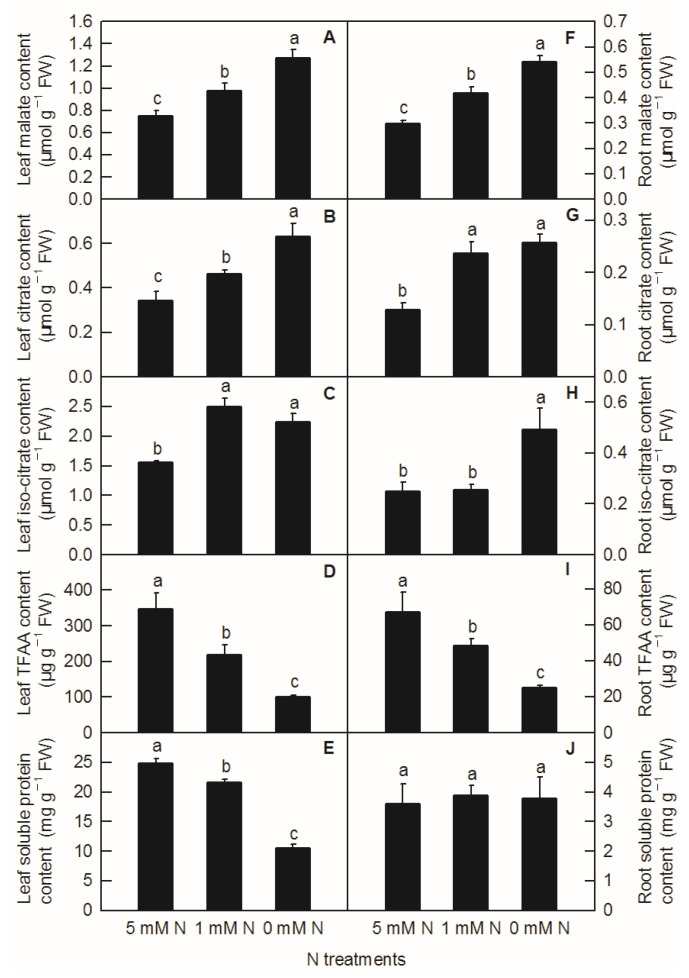
Effects of N deficiency on the contents of organic acids, TFAA, and soluble proteins in *O. sativa* leaves (**A**–**E**) and roots (**F**–**J**). Bars represent means ± SD (*n* = 3). Difference among the treatments was analyzed by Duncan’s multiple range test. Different letters above the bar indicate a significant difference at *p* < 0.05.

Different N levels affected the organic acid content of rice leaves and roots. The contents of malate, citrate in leaves, and malate in roots increased significantly with decreasing N supply (Figure 3A,B,F). Compared to the control, both low N and N deficiency significantly increased the contents of leaf isocitrate (Figure 3C) and citric acid in roots (Figure 3G), while the contents of isocitrate in roots were significantly higher under N deficiency than those under low nitrogen and control treatment (Figure 3H). With the reduction in N supply, the contents of leaf TFAA (Figure 3D), leaf-soluble protein (Figure 3E), and root TFAA (Figure 3I) showed a significant downward trend, while the content of root-soluble protein had no significant difference among different N treatments (Figure 3J). All the metabolites measured above were notably lower in the root than in the shoot (Figure 3). Furthermore, it can be seen from the above results that reducing the N supply increased the contents of malate, citrate, and isocitrate in rice leaves and roots, while it decreased the contents of TFAA and leaf-soluble protein in rice, which indicated that the change trend of organic acids and TFAA content in response to reduced N availability was similar between rice leaves and roots.

### 2.3. Effects of Different NTreatments on the Activities of Enzymes Related to Organic Acid Metabolism in Rice Leaves and Roots

**Figure 4:**
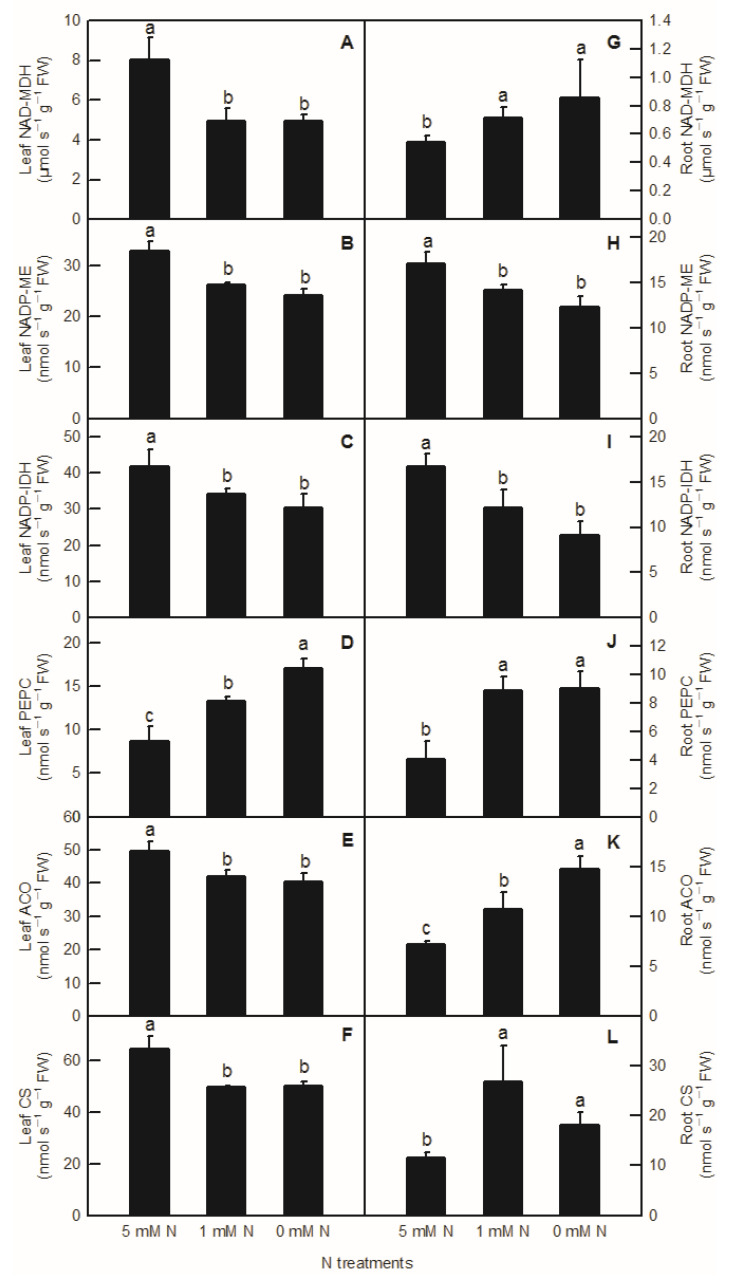
Effects of N deficiency on the activities of enzymes related to organic acid metabolism in *O. sativa* leaves (**A**–**F**) and roots (**G**–**L**). Bars represent means ± SD (*n* = 3). Difference among the treatments was analyzed by Duncan’s multiple range test. Different letters above the bar indicate a significant difference at *p* < 0.05.

Compared to the control, the activities of NAD-MDH, NADP-ME, NADP-IDH, ACO, and CS in rice leaves were significantly decreased by both low-N and N-deficiency treatments (Figure 4A–C,E,F), but their activities were not significantly different between low-N and N-deficiency treatments. With the decrease in nutrient liquid nitrogen concentration, the PEPC enzyme activity of rice leaves showed a significant upward trend (Figure 4D). In rice roots, compared to the control, low-N and N-deficiency treatments significantly increased the enzyme activities of NAD-MDH, PEPC, and CS in the root system (Figure 4G,J,L), and significantly decreased the enzyme activities of NADP-ME and NADP-IDH in rice roots (Figure 4H,I). At the same time, there was no significant difference in NAD-MDH, PEPC, CS, NADP-ME, and NADP-IDH activities between low-N and N-deficiency treatments in rice roots (Figure 4G–J,L). With the decreased N supply, root ACO showed a significant upward trend (Figure 4K). Interestingly, all the activities of enzymes related to organic acids metabolism measured above were notably lower in the root than in the shoot (Figure 4).

### 2.4. Effects of Different N Treatments on the Activities of Enzymes Related to N Metabolism in Rice Leaves and Roots

**Figure 5:**
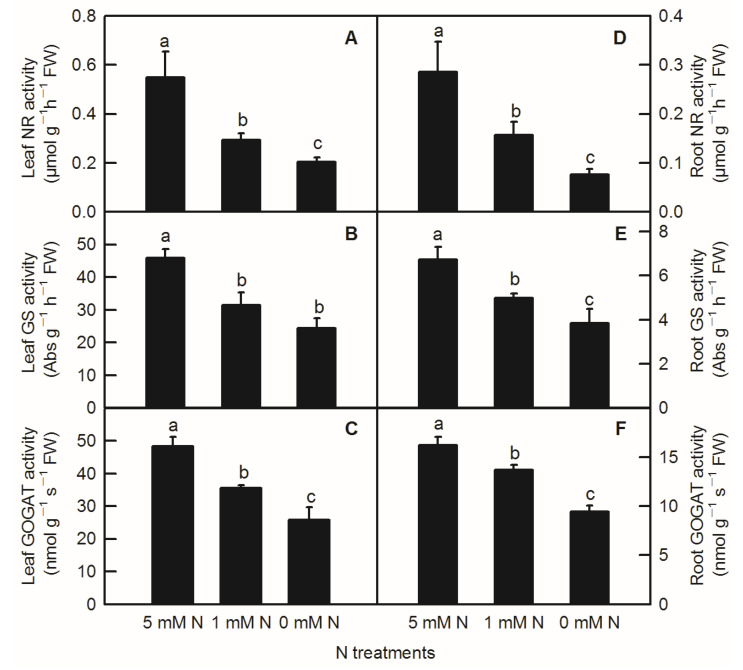
Effects of N deficiency on the activities of enzymes related to N metabolism in *O. sativa* leaves(**A**–**C**) and roots (**D**–**F**). Bars represent means ± SD (*n* = 3). Difference among the treatments was analyzed by Duncan’s multiple range test. Different letters above the bar indicate a significant difference at *p* < 0.05.

Compared to the control, N-deficiency and low-N treatment significantly reduced the activities of leaf NR, leaf GS, leaf GOGAT, root NR, root GS, and root GOGAT inrice seedlings (Figure 5). In addition to no significant change in enzyme activity of leaf GS between low-N and N-deficiency treatments, low-N treatment significantly decreased the activities of the above several enzymes related to nitrogen metabolism (Figure 5B). Similar to the parameters measured above, all the activities of enzymes related to N metabolism measured above were notably lower in the root than in the shoot (Figure 5).

### 2.5. CorrelationAnalysis and Principal Component Analysis (PCA) Loading Plots

**Figure 6:**
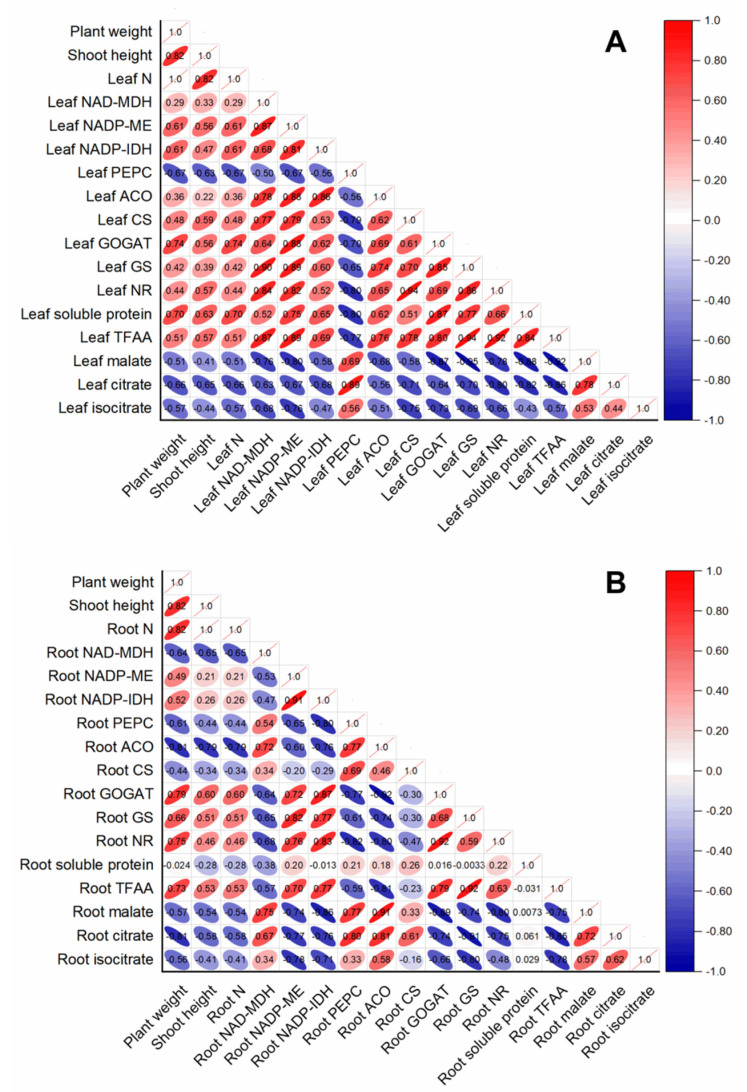
Correlation analysis of variables in rice leaves (**A**) and root (**B**) in response to different N treatments. The direction and color of the ellipse represent the positive or negative relationship between two variables. The number in the ellipse indicates the correlation coefficient between two variables.

The contents of leaf organic acids (malate, citrate, and isocitrate) were negatively correlated with all the parameters measured in rice leaf, whereas they were positively correlated with leaf PEPC (Figure 6A). Except for leaf PEPC, the activities of leaf-organic-acids-metabolism-related enzymes were all positively correlated with leaf-N-metabolism-related parameters (leaf N, leaf-soluble protein, leaf TFAA, leaf GOGAT, leaf GS, and leaf NR) (Figure 6A). In contrast, root-N-metabolism-related parameters, such as root GOGAT, root GS, root NR, and root TFAA, were positively correlated with enzymes related to root organic acids catabolism, such as root NADP-ME and NADP-IDH, whereas they were negatively correlated with enzymes related to root organic acids biosynthesis (such as root PEPC and NADP-MDH) (Figure 6B). Interestingly, root-soluble protein had no obvious correlation with other parameters measured in this study in rice root (Figure 6B).

**Figure 7:**
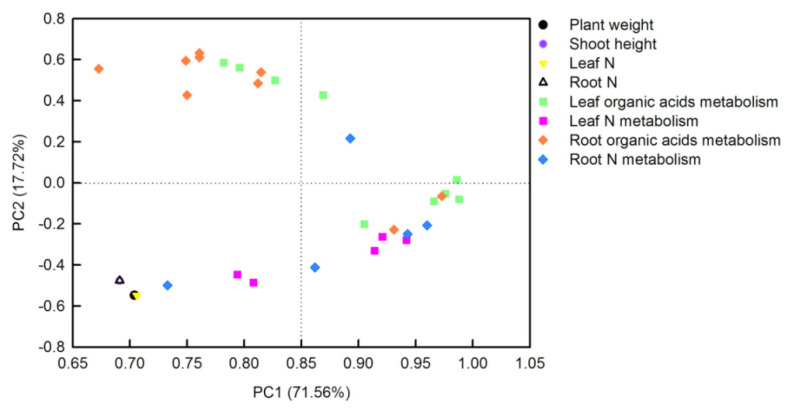
Principal component analysis (PCA) loading plot of the parameters in *O. sativa* leaves sand roots under different N levels. Thirty-two parameters from *O. sativa* leaves and roots were transformed for PCA analysis. The first two PCs explained 89.28% of the physiological variation in response to different N-supplying levels.

The principal component analysis (PCA) loading plot generated by Sigmaplot 10.0 visualized two loadings against each other to investigate the relationships between the variables. There were three or four replicates for each treatment in PCA. Thirty-two parameters including plant weight, shoot height, tissue N contents, soluble proteins, TFAAs, organic acids, and the activities of their metabolism-related-enzymes, from *O. sativa* leaves and roots, were transformed for PCA analysis (Figure 7). The first two PCs explained 89.28% of the physiological variation in response to different N-supplying levels with PC1 being accounted for by71.56% and PC2 by 17.72%. The factor loadings are listed in Table S1. The PCA result clearly showed that tissue N contents, plant weight, and shoot height were clustered tightly. Parameters related to leaf organic acids metabolism, leaf N metabolism, and root N metabolism were clustered together, whereas parameters related to root organic acids metabolism closely clustered with leaf organic acids metabolism. Furthermore, the interconnection between leaf organic acids metabolism and leaf N metabolism was closer than that between root organic acids metabolism and root N metabolism (Figure 7).

## 3. Discussion

Nfertilizer is an important factor for increasing yield in agricultural production. Both the previous literature and results of the current study showed that N deficiency could lead to the degradation of photosynthetic pigments in rice leaves and the yellowing of leaves, which eventually led to the reduction in photosynthetic rate and affected plant growth (Figure 1 and Figure 2) [2]. The phenomenon that N deficiency induceda reduction in photosynthetic rate and, thus, affected plant growth has been reported in many crops, such as tea, citrus, maize, cucumber, sunflower, tobacco, and barley [2,8,9,10,11]. According to the statistics of the Food and Agriculture Organization of the United Nations (FAO), the amount of agricultural nitrogen fertilizer has increased several times in recent decades (FAOSTAT) [29]. With the prominent increase in global population and the decline in arable land, the demand for fertilizer will increase [30]. However, excessive application of fertilizer will lead to eutrophication of the atmosphere and water system, and cause severe environmental problems. Therefore, it is of great significance to evaluate the physiological and biochemical reactions of crops to the nutrient in fertilizers to guide the rational application of fertilizers.

It was found on the model plant *Arabidopsis thaliana* that C/N interaction could significantly affect the differential expression of more than 300 genes (*p* < 0.05) [5]. Among them, the organic acid metabolic enzyme gene *PEPC* was considered to play an important role in balancing C and N metabolism [17]. In the study of transcriptomic analysis inrice under N-deficiency, Shao et al. reported that N deficiency upregulated the expression of the PEPC gene in rice leaves and increased the content of malate in rice leaves [2]. In this study, we found that with the decrease inN level, the contents of malate and citrate increased significantly in rice leaves and roots (Figure 3A,B,F). Compared to the control, both low N and N deficiency significantly increased the content of isocitrate in rice leaves and roots, while the content of root isocitrate was significantly higher under N-deficiency treatment than that under low-N and the control treatment (Figure 3C,G,H). Through the determination of the activities of organic-acid-metabolism-related enzymes, it was found that the increase in malate in rice leaves caused by the reduction inN supply was related to the increased biosynthesis of malate and the decreased degradation of malate, which was consistent with the increased PEPC activity and the decreased NADP-ME activity in rice leaves (Figure 4B,D and Figure 6A). Organic acids are synthesized by photosynthetic products through glycolysis and the TCA cycle, while 2-ketoglutarategenerated by citrate degradation in the cytoplasm is mainly used for NH_4_^+^assimilation in leaf cells [31,32]. Masumoto et al. reported that *Osppc4* was obtained by the knockdown of a leaf PEPC gene in rice and their comparative analysis of the leaf metabolome showed that *Osppc4* knockdown greatly reduced the content of organic acids, especially malate, thus inhibiting NH_4_^+^assimilation and subsequent amino acids biosynthesis in the GS/GOGAT cycle, resulting in the slowdown of plant growth [18]. In the current study, the increased contents of citrate and isocitrate in rice leaves caused by N reduction might not be caused by the increased activity of biosynthesis, but due to the reduced degradation of citrate and isocitrate in the TCA cycle, because, compared to the control, the enzyme activities of CS, ACO, and NADP-IDH in rice leaves under nitrogen reduction treatment all showed a downward trend (Figure 4C,E,F and Figure 6A). Similarly, it was also reported that N deficiency led to an increase in the contents of organic acids in potato shoot [33]. As the enzyme activities of NAD-MDH and PEPC increased in rice roots under N deficiency conditions, while the activities of NADP-ME enzymes decreased, the increased content of malate in rice roots caused by low-Nand N-deficiency treatments might be related to the increase in synthesis and reduction in degradation of malate (Figure 3F, Figure 4J,H and Figure 6B). Compared to the control, the increased content of root citrate under low N and N deficiency might be related to the increased biosynthesis of citrate rather than the decreased degradation ofcitrate, because the activities of root CS and ACO were higher in low-N and N-deficiency treatments than that of the control ones (Figure 3G and Figure 4K,L). At the same time, the root isocitrate content and root ACO activity were higher under N-deficiency treatment than those under control and low-N treatment ones, while theactivity of root NADP-IDH under N-deficiency and low-N treatment was higher than that of the control ones, which indicated that the increased isocitrate content in roots was related to the increase in citrate isomerization activity and the decrease in isocitrate degradation (Figure 3H, Figure 4I,K and Figure 6B). It can be seen from the above results that although the change mode of organic acids in rice leaves and roots under reducing N level was basically the same, the change pattern of organic-acids-metabolism-related enzymes caused by reducing N level was different between rice leaves and roots. Our correlation analysis and PCA result showed that parameters related to leaf organic acids metabolism and root organic acids metabolism were tightly clustered together, indicating that organic acids metabolism in the leaf and root were closely interconnected (Figure 6 and Figure 7).

Under N-deficient conditions, the content of ammonium used for amino acids biosynthesis in plants decreased. Therefore, the condensation of the amino and transamination process will decrease, which will inevitably lead to a change in the content of amino acids and proteins in plants [10,25]. In the current study, except for the soluble protein in rice roots, the contents of leaf TFAA, leaf-soluble protein, and root TFAA were decreased by the decreasing N level (Figure 3D,E,I,J). This is consistent with the results obtained in *Citrus* [10,25], tomato [34], tea [9], *Japonica* rice [4], and hybrid rice [35]. NR, GS, and GOGAT are three key enzymes for N metabolism in plants [36]. After nitrate is absorbed by roots, it is first reduced to nitrite by NR in the cytoplasm, and then reduced to NH_4_^+^ by nitrite reductase (NiR) in the plastid. Through the GS/GOGAT cycle, NH_4_^+^ produced from nitrate reduction and ammonium absorbed by rice ammonium transporters *Os*AMTs are assimilated into amino acids [27]. With the decreasing N level in nutrient solution, the activities of N-metabolism-related enzymes such as NR, GS, and GOGAT in rice leaves and roots decreased (Figure 5). Xiong et al. showed N deficiency at the tillering stage and N compensation at the spike differentiation stage, and N deficiency treatment could reduce the enzyme activities of NR and GS in the double cropping hybrid rice, while the normal N application and double N compensation groups increased the activities of NR and GS to a certain extent, thereby improving the photosynthetic performance of hybrid rice leaves and rice yield [35]. N deficiency treatment reduced the content of total N, ammonium, glutamine, and TFAAs in potato roots, and reduced the expression of N-metabolism-related genes such as *AMT* (ammonium transporter gene) and *GS* [34]. Similarly, Chen et al. and Huang et al. found that with the reduced N-supplying level in nutrient solution, the activities of NR, GS, GOGAT, and other enzymes related to N metabolism in *Citrus* leaves and roots decreased, resulting in a reduction in total free amino acid and soluble protein content in seedlings, and ultimately resulting in the inhibition of plant growth [10,25]. Interestingly, our PCA result demonstrated that leaf organic acids metabolism had a closer relationship with leaf N metabolism than those between root organic acids metabolism and root N metabolism (Figure 7; Table S1).

## 4. Materials and Methods

### 4.1. Plant Material and Treatment

The plant material used in this experiment was rice (*Oryza sativa* L. ssp. Indica) variety “Huanghuazhan” selected by the Rice Research Institute of Guangdong Academy of Agricultural Sciences. Uniform seeds of rice seeds were sowed in the plastic tray containing paddy soil with the content of alkaline hydrolysis N of 143 ± 6.98 mg/kg. When the height of the aboveground part of the rice seedlings was about 11.18 cm, the rice seedlings were transplanted to the Hogland nutrient solution containing 5 mM NH_4_NO_3_ (control), 1 mM NH_4_NO_3_ (low N), and 0 mM NH_4_NO_3_ (N deficiency), and cultured in a light incubator (28 °C). Seedlings were kept under a 14 h light/10 h dark regime with white photo-illumination of 150 μmol m^−2^ s^−1^ as well as a relative humidity of 68%. The nutrient solution was changed every two days. The formula of Hoagland nutrient solution contained the following macroelements (in mM): NH_4_NO_3_, 5 mM; KH_2_PO_4_, 1 mM; MgSO_4_, 2 mM; microelements (in μM):H_3_BO_3_,5 mM; MnCl_2_, 2 μM; ZnSO_4_, 2; CuSO_4_, 0.5 μM; (NH_4_)_6_Mo_7_O_24_, 0.065 μM; FeSO_4_-EDTA, 20 μM. In order to better promote the growth of rice seedlings, a concentration of 0.1 μM Na_2_SiO_3_ was added to each nutrient solution. After 15 days of culture, leaf and root samples were collected to determine the fresh weight and height of shoots after the phenotype of seedlings were recorded (Figure 1). There were four replicates for fresh weight and shoot height. At the same time, the rice plants were divided into upper and lower parts with scissors and put into aluminum foil bags. After freezing in liquid nitrogen, allthe samples were stored at −80 °C until assayed.

### 4.2. Determination of N Content in Rice Leaves and Roots

The nitrogen content was determined by the Kjeldahl method [2]. The ground samples of rice leaves and roots were digested with concentrated H_2_SO_4_-H_2_O_2_, and then the N contents in rice leaves and roots were analyzed using a Foss Kjeltec 8200 nitrogen analyzer (Hilleroed, Denmark). There were four replicates for the measurement of leaf N and root N.

### 4.3. Determination of Organic Acids, Total Free Amino Acids (TFAA),and Total Soluble Proteins in Rice Leaves and Roots

The extraction and determination of organic acids in rice leaves and roots were carried out according to the method described by Lu et al. [37]. Organic acids were extracted by 4% perchloric acid and centrifuged to determine the contents of malate, citrate, and isocitrate. The reaction mixture of malate contained 50 mM 3-amino-1-propanol-HCl (pH = 10), 30 mM sodium glutamate-NaOH (pH = 10), 2.7 mM NAD, 1 Uglutamate-oxaloacetate transaminase (GOT, EC 2.6.1.1), 10 U NAD-malate dehydrogenase (NAD-MDH, EC 1.1.37), and 50 µL of extracted supernatant. The reaction system of citrate contained 100 mM Tris-HCl (pH = 7.6), 0.2 mM NADH, 7 U lactate dehydrogenase (LDH, EC 1.1.1.27), 14 U NAD-MDH, 0.5 U citrate lyase (EC 4.1.3.6), and an appropriate amount of extracted supernatant. The reaction system of isocitrate contained 100 mMTris-HCl (pH = 7.6), 3.3 mM MnSO_4_, 0.2 mM NADP, 0.1 U NADP isocitrate dehydrogenase (NADP-IDH, EC 1.1.1.42), and an appropriate amount of extracted supernatant. There were three biological replicates for each treatment.

TFAAs were determined according to the method of Li et al. [38]. The total free amino acids were determined by the ninhydrin method. About 0.1 g of root and leaf samples were ground and extracted with 1.6 mL of 10% acetic acid. After centrifugation at 12,000× *g* for 10 min at 4 °C, 0.9 mL of ultrapure water, 1.5 mL of hydrated ninhydrin solution, and 0.25 mL of 1% ascorbic acid were added to a 5 mL reaction tube containing 0.1 mL of supernatant, mixed well, and reacted in a boiling water bath for 15 min. After reaction, 1.25 mL of 60% ethanol was added to the mixture, and after mixing, the absorbance value at 570 nm wavelength was measured with an Ultraviolet/Visible spectrophotometer. Leucine was used to make the standard curve and the concentration of leucine for the standard curve ranged from 0 µg/mL to 2.5 µg/mL. Each treatment was performed for three biological replicates. The total soluble protein content was determined by the Coomassie brilliant blue method described by Bradford [39]. Rice samples (about 0.1 g) were extracted by grinding with 1.6 mL of 50 mM Na_2_HPO_4_-KH_2_PO_4_ (pH = 7.0) and centrifuged at 3000× *g* for 10 min at 4 °C. Twenty-five microliters of supernatant and 975 μLof Coomassie brilliant blue solution were mixed and allowed to react at 25 °C for 5 minutes, and then the absorbance at 595 nm was determined. At the same time, bovine serum albumin (BSA) was used to prepare standard curves under the same conditions, and each treatment had three biological replicates.

### 4.4. Determination of Enzymes Related to Organic Acid Metabolism and N Metabolism in Rice Leaves and Roots

The extraction and activity measurement of organic-acid-metabolism-related enzymes in rice leaves and roots were carried out according to the method described by Lu et al. [37]. The extraction solution contained 50 mM HEPES KOH (pH = 7.5), 10 mM MgCl_2_, 2 mM EDTA-Na_2_, 10 mM dithiothreitol (DTT), 1% (*v/v*) Triton X-100, 5% water-insoluble PVPP, 1% (*w/v*) BSA, and 30% glycerol. About 0.1 g of leaf or root samples was ground with 1.6 mL of extraction solution. The tissue homogenate (about 1.7 mL) was centrifuged at 13,000× *g* for 5 min, and the supernatant was used for enzyme activity measurement.

The assay of the enzyme related to N metabolism in rice was carried out according to the method described by Hageman et al. [40]. The extraction solution of NR contained 10 mM cysteine, 1 mM EDTA-Na_2_, 5% PVPP, and 25 mM phosphate buffer (pH = 8.7). Approximately 0.1 g of rice leaf or root samples was added to pre-cooled mortar containing a small amount of quartz sand and the above phosphate buffer for grinding and extraction. The homogenate was centrifuged at 4000× *g* for 15 min at 4 °C. The reaction mixture for NR activity contained 0.2 mL of extract supernatant, 100 mMKNO_3_, and 2 mMNADH. The reaction mixture was incubated in a 30 ℃ water bath for 30 min. At the end of the reaction, 1% (*w/v*) sulfonamide was added immediately to terminate the reaction, and then 0.02% (*w/v*) naphthylamine solution was added to the mixture for reaction at 30 °C for 15 min. The total volume of the reaction mixture was 2 mL. Finally, the absorbance of the mixed solution was measured at 540 nm, and a blank control was set for each sample. NaNO_2_ was used to make the standard curve, and the concentrations for the curve ranged from 0.01 μg/mL to 0.1 μg/mL of NO_2_^−^.

GS and GOGAT extraction solution contained 100 mM Tris HCl (pH = 7.5), 1 mM MgCl_2_, 1 mM EDTA-Na_2_, 1mM β-mercaptoethanol, 0.3% Triton X-100, and 5% PVPP. After grinding and centrifugation at 16,000× *g* 4 °C for 30 min, the supernatant was taken for enzyme determination. The GS activity measurement mixture contained a mixture of 80 mM Tris HCl, 40 mM MgCl_2_, 40 mM sodium glutamate, 16 mM hydroxylamine hydrochloride, and 8 mM ATP. The mixed solution was measured to react for 30 minutes at 30 °C in a water bath. After the reaction, a chromogenic agent containing 2% TCA solution (*w/v*), 3.5% FeCl_3_ (*w/v*), and 1.8% HCl (*v/v*) was added to the mixture. After the reaction for 5 min, the reaction mixture was centrifuged at 10,000× *g* for 5 min, and the supernatant was taken to measure its absorbance at 540 nm. A blank control was set for each sample. The reaction system for GOGAT determination contained 100 mM2-ketoglutarate, 10 mM KCl, 3 mM NADH, and 25 mM Tris HCl (pH = 7.6), and mixed well. Finally, 20 mM glutamate was added to start the reaction, and the enzymatic kinetic reaction was measured at 340 nm for 60 s.

### 4.5. Experimental Design and Statistic Analysis

In this experiment, three culture pots were set for each N treatment, and each culture pot contained 18 rice seedlings. Duncan’s multiple range test was used for variance analysis between different treatments. Correlation plots were generated by Origin software (Version: Pro 2022b SR1). The experimental data and PCA were processed by Microsoft Excel 2010, analyzed by SPSS 19.0, and visualized by Sigmaplot 10.0 [41].

## 5. Conclusions

In this study, we found that low N and N deficiency reduced rice biomass, plant height, and N content, increased the content of main organic acids, and decreased the total free amino acids and leaf-soluble proteins in *O. sativa*. Although the dynamics of the contents of organic acids in rice leaves and roots were basically the same under low N and N deficiency, the dynamics of the activities of organic-acid-metabolism-related enzymes caused by reducing N-supplying level were different in rice leaves and roots.

With the decreased N-supplying level, the activities of N-metabolism-related enzymes, such as NR, GS, and GOGAT, decreased in rice leaves and roots, resulting in the decreased contents of TFAAs and total soluble protein, and finally resulting in the inhibition of plant growth. The signal of N deficiency might not directly act on the TCA cycle, but on the organic acid biosynthesis step in the cytoplasm or the upstream transcriptional regulation level.

The dynamics of organic acids metabolism caused by N deficiency were different in rice leaves and roots. Interestingly, our PCA result demonstrated that leaf organic acids metabolism had a closer relationship with leaf N metabolism than that between root organic acids metabolism and root N metabolism. In the future study, more effort should be focused on the specific molecular and physiological mechanism underlying how N deficiency regulates organic acids metabolism in rice plants.

## Data Availability

Not applicable.

## Supplementary Materials

The following supporting information can be downloaded at: https://www.mdpi.com/article/10.3390/plants11192576/s1, Table S1: Factor loadings of variables of first two principal components.
